# Laparoscopic transabdominal preperitoneal repair for female patients with groin hernias

**DOI:** 10.1186/s12905-023-02527-5

**Published:** 2023-08-09

**Authors:** Ronggui Lin, Xianchao Lin, Yuanyuan Yang, Congfei Wang, Haizong Fang, Yanchang Chen, Heguang Huang, Fengchun Lu

**Affiliations:** Department of General surgery, Fujian Medical University Union Hospital, Fuzhou, Fujian China

**Keywords:** Groin hernia, Round ligament of the uterus, Female, Laparoscopic repair

## Abstract

**Background:**

Laparoscopic transabdominal preperitoneal repair (TAPP) was recommended for female patients with groin hernias. Whereas, only a few studies focused on whether and how to preserve the round ligament of the uterus in TAPP.

**Methods:**

Clinical data of 159 female patients with 181 groin hernias who underwent TAPP at a single institution in China from January 2016 to June 2022 were retrospectively reviewed and collected.

**Results:**

All the patients underwent the operation smoothly without conversion. Division of the round ligament was performed for 33 hernias. Preservation of the round ligament was adopted for 148 hernias, 51 with the “keyhole” technique, 86 with the “longitudinal incision of peritoneum” method, and 11 with total dissection of the round ligament. The mean operative time was 55.6 ± 8.7 min for unilateral TAPP and 99.1 ± 15.8 min for bilateral TAPP. The mean estimated blood loss was 7.1 ± 4.5 mL. The postoperative complications included 6 (3.3%) cases of seroma, 1 (0.5%) case of hematoma, and 3 (1.6%) cases of mild chronic pain. The incidences of chronic pelvic pain and genital prolapse seemed to be higher in the division group than in the preservation groups (6.1% vs. 0.6% and 3.0% vs. 0%, separately). However, no statistical difference was observed (p > 0.05).

**Conclusions:**

TAPP is feasible and effective for female patients with groin hernias, especially in preserving the round ligament of the uterus.

**Supplementary Information:**

The online version contains supplementary material available at 10.1186/s12905-023-02527-5.

## Introduction

The incidence of groin hernia in female patients is lower than that in male patients [1]. It accounts for approximately 8% of groin hernia in the Swedish Hernia Registry [2]. The rate of emergency operation for female patients, at 14.5–17.0%, is 3 to 4-fold higher than that of male patients [3]. For female patients, femoral hernias have a higher incidence of incarceration and strangulation than inguinal hernias, with a rate of emergency operation of 40.6% [3]. Therefore, timely repair is suggested for female groin hernias in European guidelines by the HerniaSurge Group [4].

Laparoscopic repair, including transabdominal preperitoneal repair (TAPP) and totally extraperitoneal repair (TEP), was recommended as the method of choice for female patients with groin hernia if expertise is available in the international guidelines on the management of groin hernias [4]. For open repair procedures, the femoral canal is not routinely explored in Lichtenstein procedure, which could result in a “false” recurrence with a missed femoral hernia. Laparoscopic preperitoneal repair provides a thorough view and exploration of the myopectineal orifice to avoid missing a femoral hernia [5, 6]. Recurrent inguinal hernia repair in female patients was usually diagnosed with a femoral hernia during reoperation [7]. Meanwhile, laparoscopic preperitoneal repair covers the three defect areas of groin hernias with a low incidence of recurrence [8]. Laparoscopic preperitoneal repair has fewer postoperative complications than open repair [9]. Hence, TAPP or TEP is recommended for female groin hernia repair.

According to the technical aspect of laparoscopic preperitoneal repair for female patients, controversy exists about whether to divide or preserve the round ligament of the uterus. The round ligament of the uterus runs from the anterior part of the uterus through the inguinal canal and terminates in the labium majorum [10]. It helps maintain the anteverted position of the uterus, though it is not considered essential for uterus suspension. However, concerns were raised about the possible complications associated with the division of the round ligament in groin hernia repair.

In this study, we performed TAPP for 159 female patients with 181 groin hernias. Three techniques were used to preserve the round ligament of the uterus during TAPP with satisfactory postoperative outcomes.

## Methods

Electronic medical records of female patients with groin hernias who underwent TAPP at a single institution from January 2016 to June 2022 were retrospectively reviewed and collected. Groin hernias were diagnosed by the presence of bulging in the groin area and were confirmed by ultrasonography.

Demographic data including age, body mass index (BMI), hernia location, hernia classification, hernia type, and perioperative data including repair techniques, operative time, estimated blood loss, visual analogue scale (VAS) on postoperative day 1 (POD 1), length of stay and postoperative complications were collected and analyzed. Chronic pelvic pain refers to chronic pain in the pelvic area which lasts longer than 3 months. Genital prolapse refers to uterine, uterovaginal, or vaginal prolapse [11]. Loss of support in the pelvic region is a main cause of genital prolapse.

### Operative technique

The process of TAPP procedure was previously described [12]. Briefly, a Veress needle was inserted into the abdominal cavity. CO_2_ was insufflated to establish the pneumoperitoneum with a pressure of 12–14 mmHg. A 10-mm trocar was inserted above the umbilicus, and two additional trocars were placed at the lateral edge of rectus abdominis at the horizontal level of the umbilicus.

The peritoneum was incised 2 cm above the internal ring from lateral to medial. The inferior epigastric vessels were identified, and the Retzius space was entered lateral to the medial umbilical ligament and dissected till the pubic bone was exposed. The Bogros space was separated, and the preperitoneal fascia was well preserved to avoid exposure of nerves. For direct hernias, femoral hernias and small indirect hernias, the hernia sac was totally dissected. For large indirect hernias, the hernia sac was divided at the internal ring level.

**Fig. 1:**
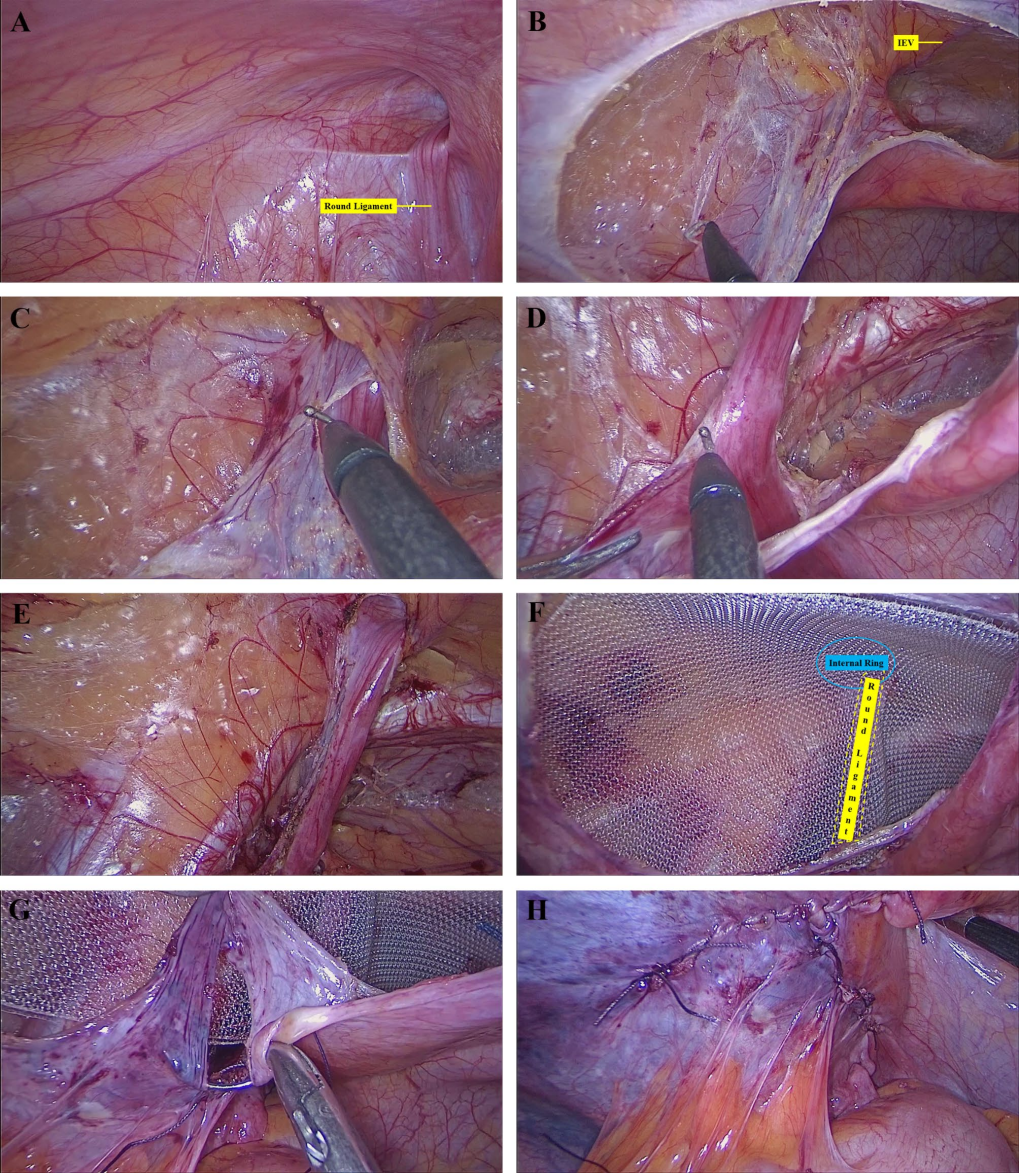
The “longitudinal incision of peritoneum” method to preserve the round ligament of the uterus in TAPP. **(A)** Exploration of a female patient with a left indirect hernia; **(B)** Dissection of Bogros space after separation of Retzius space; **(C)** Division of hernia sac at the internal ring level; **E**. Longitudinal incision of the peritoneum besides the round ligament to a high level; **F**. Separated preperitoneal space; F. Placement of a heavyweight polypropylene mesh; **G**. Closure of incised peritoneum besides the round ligament. **H**. Closure of peritoneum flap

**Fig. 2:**
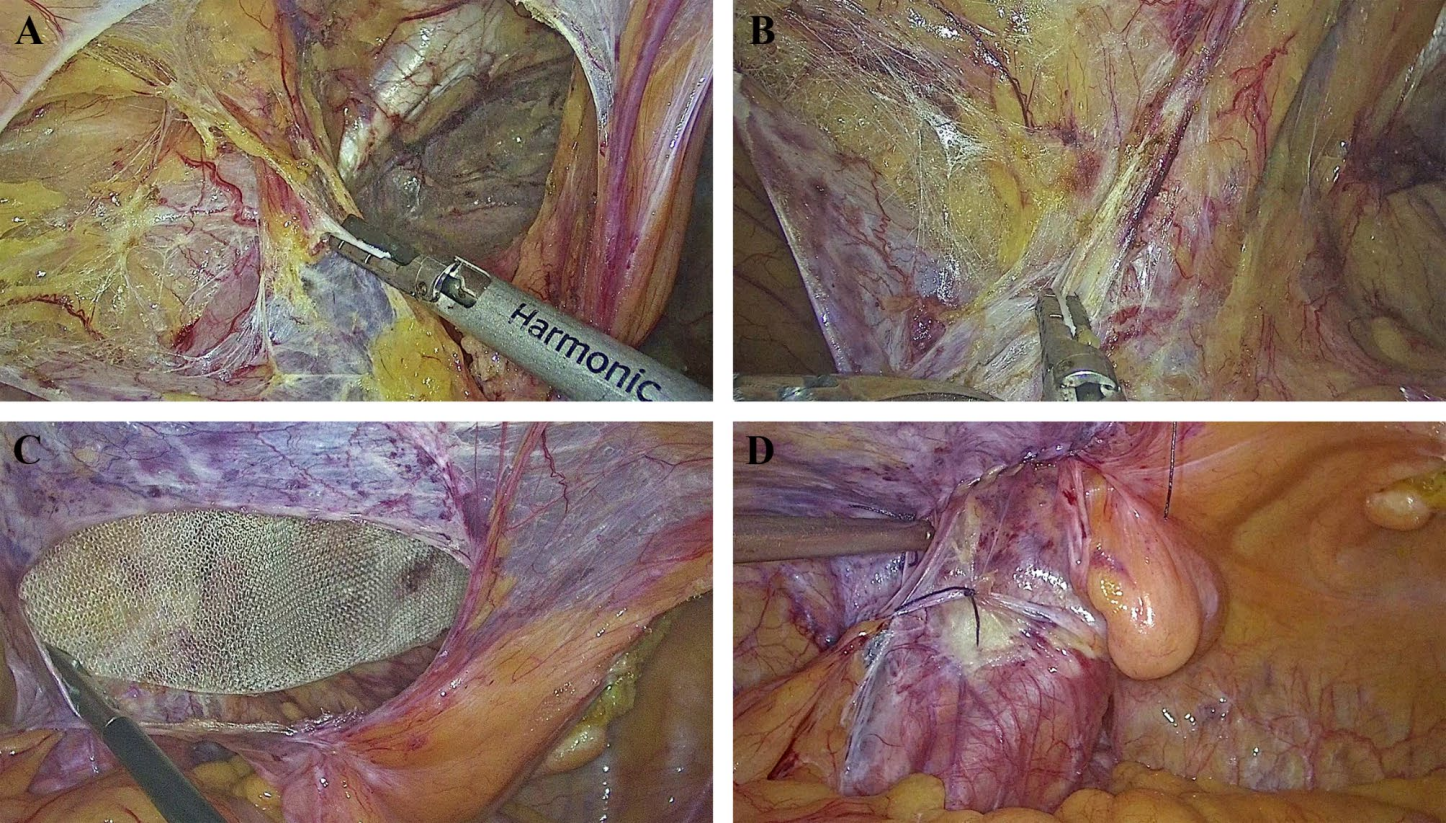
Total dissection of the round ligament of the uterus in TAPP. **A-B**. Separation of the round ligament of the uterus from the peritoneum after total dissection of hernia sac; **C**. Placement of a heavyweight polypropylene mesh; **D**. Closure of peritoneum flap

**Fig. 3:**
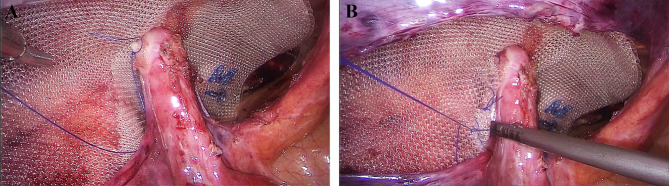
The “keyhole” technique to preserve the round ligament of the uterus in TAPP. **(A)** The incised mesh was placed into the preperitoneal space with a suture to the round ligament for fixation. **(B)** The opening of the mesh was overlapped with suture using 4 − 0 prolene to restore the integrity of the mesh

Three methods were adopted to preserve the round ligament of the uterus. ①the “longitudinal incision of peritoneum” method (Video 1): The peritoneum besides the round ligament was longitudinally incised to a high level to allow parielization of the ligament. The peritoneum was closed by suture after mesh placement (Fig. 1). ②Total dissection of the ligament: the round ligament was totally dissected if there was no dense adhesion between the peritoneum and the ligament (Fig. 2). ③the “keyhole” technique: the round ligament was preserved and the plane in front of the ligament was separated. The mesh was cut and placed around the ligament (Fig. 3). The opening of the mesh was overlapped with suture using 4 − 0 prolene thread. For the non-preservation group, the round ligament was divided and the preperitoneal space was separated in front of the ligament. To prevent injury of the genital branch of the genitofemoral nerve, the round ligament was divided at a relatively high level, before it enters the internal ring.An adequate preperitoneal space was then created and a heavyweight polypropylene mesh was placed to cover the myopectineal orifice. No tack or glue fixation was adopted. Finally, the peritoneum was closed by running suture with absorbable threads. The CO_2_ in the preperitoneal space was extracted by suction to facilitate the fixation of the mesh.

The patients returned for follow-up at outpatient service one week, three months and one year after the operation. The patients were informed to extend follow-up in the presence of clinical manifestations [11].

### Statistical analysis

Statistical analysis was performed using SPSS 23.0 software (IBM, USA). Continuous data were presented as mean ± standard deviation (SD). Categorical data were presented as percentage (%). Continuous data were compared with *t* tests or nonparametric Mann-Whitney U tests. Categorical data were compared with χ² or Fisher’s exact tests. A p value < 0.05 was considered as significant.

## Results

A total of 159 female patients with 181 groin hernias were included in this study (as indicated in Table 1). The mean age was 58.5 ± 15.2 with a mean BMI of 22.6 ± 1.3. Hernia location was 57 cases on the left, 80 cases on the right and 22 cases with bilateral hernias. There were 130 indirect hernias, 19 direct hernias and 32 femoral hernias. The hernias included 55 Type I (< 1.5 cm) hernias, 85 Type II (1.5-3.0 cm) hernias, 41 Type III (> 3.0 cm) hernias, and 15 recurrent hernias, according to the European hernia society groin hernia classification [13].

**Table 1:** Perioperative data of female patients with TAPP

Perioperative data	n
Patients/hernias	159/181
Age (years)	58.5 ± 15.2
BMI (kg/m^2^)	22.6 ± 1.3
Hernia location	
Left	57
Right	80
Bilateral	22
Hernia classification	
Indirect hernia	130
Direct hernia	19
Femoral hernia	32
Hernia type	
Type I (< 1.5 cm)	55
Type II (1.5-3.0 cm)	85
Type III (> 3.0 cm)	41
Recurrent hernia	15
Division of the round ligament	33
Preservation of the round ligament	
“Keyhole” technique	51
Longitudinal incision of peritoneum	86
Total dissection of the round ligament	11
Operative time (minutes)	
Unilateral	55.6 ± 8.7
Bilateral	99.1 ± 15.8
Blood loss (mL)	7.1 ± 4.5
VAS on POD1	2.5 ± 0.7
Length of hospital stay (days)	1.1 ± 0.2
Postoperative complications	
Seroma	6 (3.3%)
Haematoma	1 (0.5%)
Recurrence	0
Chronic groin pain	3 (1.6%)

TAPP, transabdominal preperitoneal repair; BMI, body mass index; VAS, visual analogue scale; POD1, postoperative day 1

There was no conversion to other procedures. Division of the round ligament was performed for 33 hernias, most of which were performed in the early phase. Preservation of the round ligament was adopted for 148 hernias, 51 with the “keyhole” technique, 86 with the “longitudinal incision of peritoneum” method and 11 with total dissection of the round ligament. The mean operative time was 55.6 ± 8.7 min for unilateral TAPP and 99.1 ± 15.8 min for bilateral TAPP. The mean estimated blood loss was 7.1 ± 4.5 mL. The mean VAS on POD1 was 2.5 ± 0.7 and the mean length of stay was 1.1 ± 0.2 days. The postoperative complications included 6 (3.3%) cases of seroma, 1 (0.5%) case of hematoma, and 3 (1.6%) cases of mild chronic pain. There was no recurrence or incisional or mesh infection during a mean follow-up of 37 months (3–80 months). The incidences of chronic pelvic pain and genital prolapse seemed to be higher in the division group than in the preservation group (6.1% vs. 0.6% and 3.0% vs. 0%) with a mean follow-up of 44 and 36 months, separately (Table 2). However, no statistical difference was observed (p > 0.05). No patient suffered from dyspareunia in both groups.

**Table 2:** Long-term postoperative complications of female patients with TAPP

Variables	Division group n (%)	Preservation group n (%)	*p* value
Hernias	33	148	
Follow-up period (months)	44	36	0.165
Postoperative complications			
Chronic pelvic pain	2 (6.1%)	1 (0.6%)	0.086
Genital prolapse	1 (3.0%)	0 (0.0%)	0.182
Dyspareunia	0 (0.0%)	0 (0.0%)	1.000

## Discussion

In this present study, 159 female patients with 181 groin hernias underwent TAPP. The round ligament of the uterus was divided in 33 hernias and was preserved in 148 hernias using three techniques. No severe complications occurred, indicating the safety and feasibility of three techniques for the preservation of the round ligament.

Preservation the round ligament of the uterus has not attracted much attention of general surgeons. A recent nationwide survey in Denmark indicated that personal opinions and knowledge about the function of the round ligament of the uterus and whether to preserve the ligament varied greatly among the surgeons [14]. Many surgeons demonstrated that standard surgery and correct placement of mesh were more important than the preservation of the round ligament. The survey also showed that the round ligament of the uterus was transected in an estimated half of female patients with laparoscopic groin hernia repair. A recent study enrolling 1365 female patients with open, laparoscopic, or robotic inguinal hernia repair showed that the round ligament was transected in 868 (63.6%) cases [15].

Controversy exists about whether to divide or preserve the round ligament of the uterus in TAPP or TEP. A recent comparative study showed that division or preservation of the round ligament of the uterus in TEP had no statistically significant differences in terms of chronic pain, uterine prolapse or paresthesia [16]. A retrospective study enrolled 218 female patients with preservation of round ligament and 175 female patients with transection of round ligament during laparoscopic inguinal hernia repair [17]. No obvious effect on subsequent pregnancy or childbirth was noticed during a long-term follow-up of 42 months. No difference in dyspareunia, chronic pelvic pain, dysmenorrhea, or uterine prolapse. A nationwide linked register-based cohort study in Denmark showed no difference in genitourinary complications between open and laparoscopic groin hernia repair in women [18]. The urogenital consequences of transection of the round ligament of the uterus seemed to be minimal. However, the potential consequence remains blurred because of limited evidence. Concern was also raised that transection of the round ligament of the uterus in inguinal hernia repair may result in a retroflexed uterus, which might be associated with sexually related pain, chronic pelvic pain, dysmenorrhea, or even uterus prolapse [19]. It is of great importance for young female patients, especially for those with fertility requirements in the future [20].

During embryological development, the caudal part of the female gubernaculum forms the round ligament of the uterus, connecting the uterine horns to the labia majora via the inguinal canal. The round ligament of the uterus is covered by folded peritoneum and forms the superior margin of the broad ligament of the uterus [21]. Therefore, division of the round ligament of the uterus is common in TAPP mainly because of dense adhesion between the peritoneum and the ligament. However, the surgeon would be willing to preserve the round ligament if there were effective methods. Three methods of preserving the round ligament were described in detail in this study. First, the round ligament was dissected from the peritoneum to a great extent as parielization of spermatic vessels and vas deference in male patients. This method is suitable for only a few female patients with a separable round ligament. Second, the peritoneum besides the round ligament was incised until a high level of parielization and was closed after mesh placement. This method is suitable for many female groin hernias and is now the first choice to preserve the round ligament. Whereas, it is unsuitable for some shortened and swollen round ligaments with uterine deviation toward the hernia side [22]. Third, the keyhole method was adopted which allowed the ligament to pass through and the openings of the mesh were overlapped with suture using 4 − 0 prolene threads. It is suitable for most of the types of female groin hernias and was mostly adopted in the early phase of TAPP to preserve the round ligament in our center.

Doubt might exist about the continuity and integrity of the mesh with the keyhole method. Tips are important for this issue. The mesh was incised in the non-defect area. As for an indirect or direct hernia, the mesh was incised at the inferomedial direction; for a femoral hernia, the mesh was incised at the superomedial direction. Meanwhile, the opening of the mesh was overlapped with suture using prolene thread instead of absorbable thread to restore the integrity of the mesh. A suture to the round ligament can also prevent mesh displacement. No recurrence was observed in this study, indicating the safety of the three methods.

TAPP has some advantages in the preservation of the round ligament of the uterus as compared to other procedures. For Lichtenstein procedure, the round ligament is easy to preserve while the femoral canal is not routinely explored with the potential risk of a missing femoral hernia. For open preperitoneal repair, such as Kugel procedure, it is feasible to repair the entire myopectineal orifice while it is difficult to preserve the round ligament of the uterus, either dissecting the round ligament from the peritoneum or suturing the incised peritoneum besides the round ligament during parielization. For TEP, the incised peritoneum significantly affects the laparoscopic view and it is difficult to suture the peritoneum or mesh with the methods mentioned above. However, laparoscopic view and operative field in TAPP are better and larger than the other procedures. It is more convenient to suture in TAPP, which is beneficial for the preservation of the round ligament.

The major limitation of the study is the retrospective analysis of patients in a single institution. A multi-center randomized control trial enrolling more patients would be required to further evaluate the three methods of preservation of the round ligament of the uterus.

## Conclusions

TAPP is feasible and effective for female patients with groin hernias with a low incidence of postoperative complications. it shows advantages in preserving the round ligament of the uterus.

### Electronic supplementary material

Below is the link to the electronic supplementary material.

**Additional File 1:** Video legend

**Additional File 2:** TAPP with preservation of the round ligament of the uterus using the “longitudinal incision of peritoneum” method

## Data Availability

The datasets used and/or analysed during the current study available from the corresponding author on reasonable request.
