# Supplementary material for: Laparoscopic transabdominal preperitoneal repair for female patients with groin hernias
Source: BMC Womens Health. 2023 Aug 9;23:422. doi: 10.1186/s12905-023-02527-5 (PMC10413729; doi:10.1186/s12905-023-02527-5)
Supplement: Supplementary file 2 — Additional File 2: TAPP with preservation of the round ligament of the uterus using the “longitudinal incision of peritoneum” method [file 12905_2023_2527_MOESM2_ESM.docx]

**Video legend**

**Video 1. TAPP with preservation of the round ligament of the uterus using the “longitudinal incision of peritoneum” method**

This video depicts a female patient with a left indirect inguinal hernia who underwent transabdominal preperitoneal repair (TAPP) with preservation of the round ligament of the uterus. The round ligament of uterus was preserved with the “longitudinal incision of peritoneum” method. After laparoscopic exploration of abdominal cavity, the peritoneum was incised 2 cm above the internal ring from lateral to medial. The Retzius space was separated followed by the separation of the Bogros space. The hernia sac was divided at a high level. The peritoneum besides the round ligament was longitudinally incised to a high level to allow parielization of the round ligament. An adequate preperitoneal space was then created and a heavyweight polypropylene mesh was placed to cover the myopectineal orifice. The peritoneum was closed by running suture with absorbable threads. The patient was discharged from hospital on postoperative day one with no issues.
